# Physiological pacing to improve cardiac resynchronization therapy non-responder and a tryst with calcified septum—a case report

**DOI:** 10.1186/s43044-021-00145-w

**Published:** 2021-03-18

**Authors:** Vanita Arora, Francesco Zanon, Viveka Kumar, Vivek Kumar, Pawan Suri

**Affiliations:** 1 Max Healthcare Superspeciality Hospital, Saket, New Delhi, India; 2 Santa Maria della Misericordia Hospital, Rovigo, Italy; 3 SGL Multispeciality Hospital, Jalandhar, Punjab India

**Keywords:** Physiological pacing, His bundle pacing, Case report, HOTCRT

## Abstract

**Background:**

As per the literature, patients with intraventricular conduction delay (IVCD) do not respond well to cardiac resynchronization therapy (CRT) alone. They need advanced technological approach and out of the box thinking for a good response.

**Case:**

Ours is a case of ischemic cardiomyopathy with wide QRS-IVCD, a non-responder to CRT. While planning for replacement of the device for early replacement indicator (ERI), we decided to do His-optimized CRT/left bundle optimized CRT (HOT-CRT/LOT-CRT) for the patient.

**Conclusion:**

The challenges we faced with the present available hardware paved a way for insisting on the limitation of the available lumenless lead to penetrate calcified the septum and importance of the pre-procedure evaluation of intraventricular septum (IVS) for calcification by more than just echocardiography.

## Background

Cardiac resynchronization therapy (CRT) has widely been used in patients of symptomatic chronic heart failure (HF) with wide QRS complex, refractory to optimal medical therapy and having left ventricular ejection fraction

LVEF is < 35%. However, one third of the patients are non-responders and do not respond to this therapy. Physiological pacing, i.e., permanent His bundle pacing (HBP)/left bundle branch pacing (LBBP) is proving a promising alternative to biventricular pacing (BiVP) for some of these challenging patients. In this report, we present a case of HOT-CRTD with good outcome of physiological pacing despite challenges.

## Case presentation

**Fig. 1:**
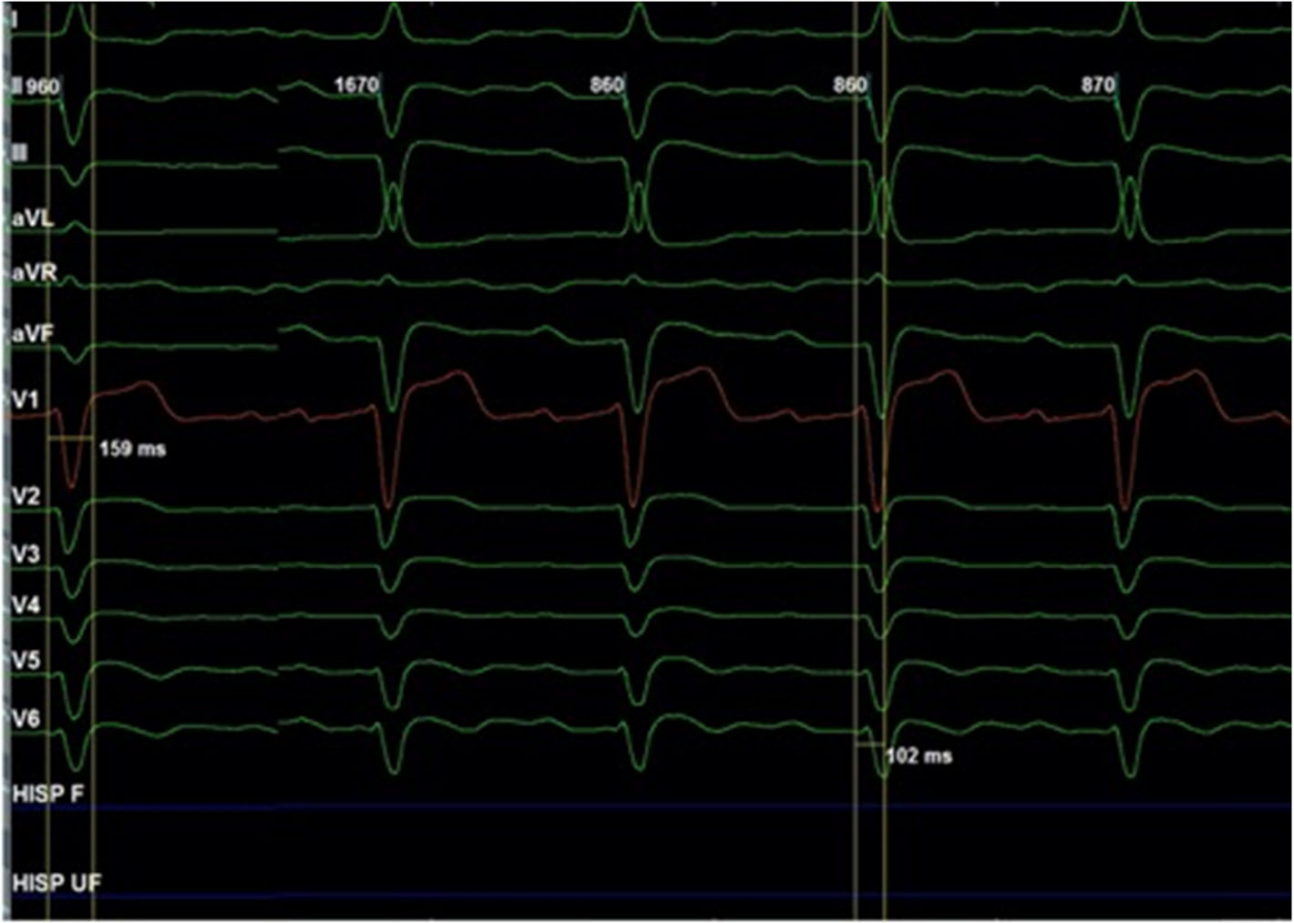
Baseline ECG with intraventricular conduction delay (IVCD) with wide QRS 159 ms, QLV of 102 ms

**Fig. 2:**
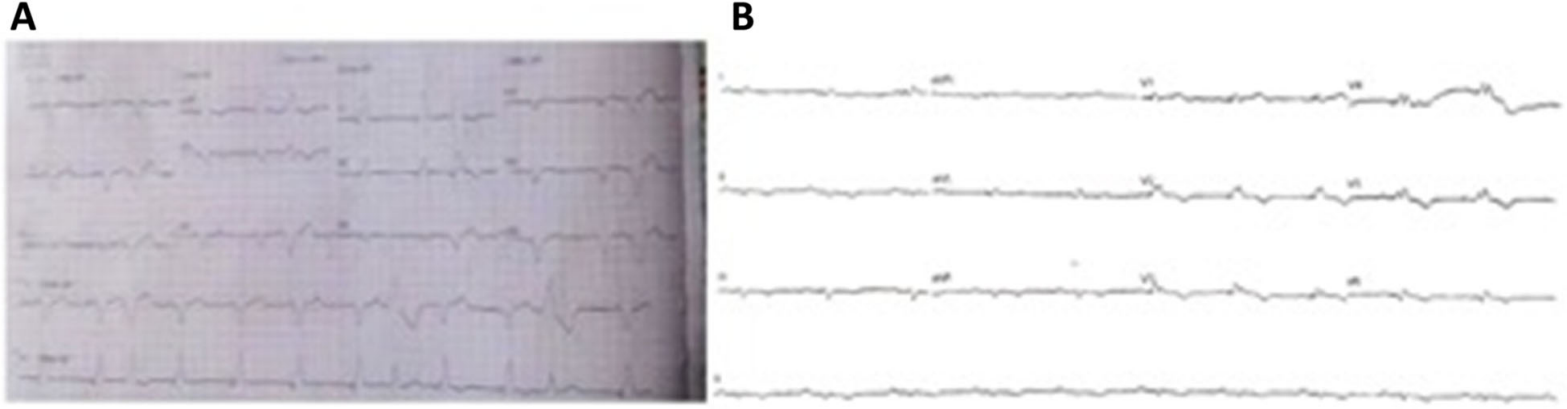
**a** Post-CRT paced ECG showed positive V1 and negative lead I with QRS duration of 160 ms. **b** Post-HBP with LV delay of 40 ms and AV delay of 80 ms, narrow (106 ms) complex QRS was achieved

A 64-year-old male patient with a known history of coronary artery disease had undergone a CRTD implant in November 2015 for IVCD with wide QRS (159 ms), QLV was 102 ms (Fig. 1) and NYHA class III-IV with LVEF 15%. The post-CRT paced ECG showed positive V1 and negative lead I with QRS duration of 160 ms (Fig. 2a). The patient remained symptomatic despite being on optimal medical therapy qualifying as a non-responder. Since the device had reached recommended replacement time (RRT) and patient continued to be symptomatic for HF in NYHA class III with an EF of 15% and pulmonary artery systolic pressure (PASP) 60 mm of Hg, an alternative technique to physiologically pace via the His bundle pacing (HBP)/left bundle branch pacing (LBBP) with appropriately timed pacing of left ventricle (LV) for a narrower fusion complex was considered.

**Fig. 3:**
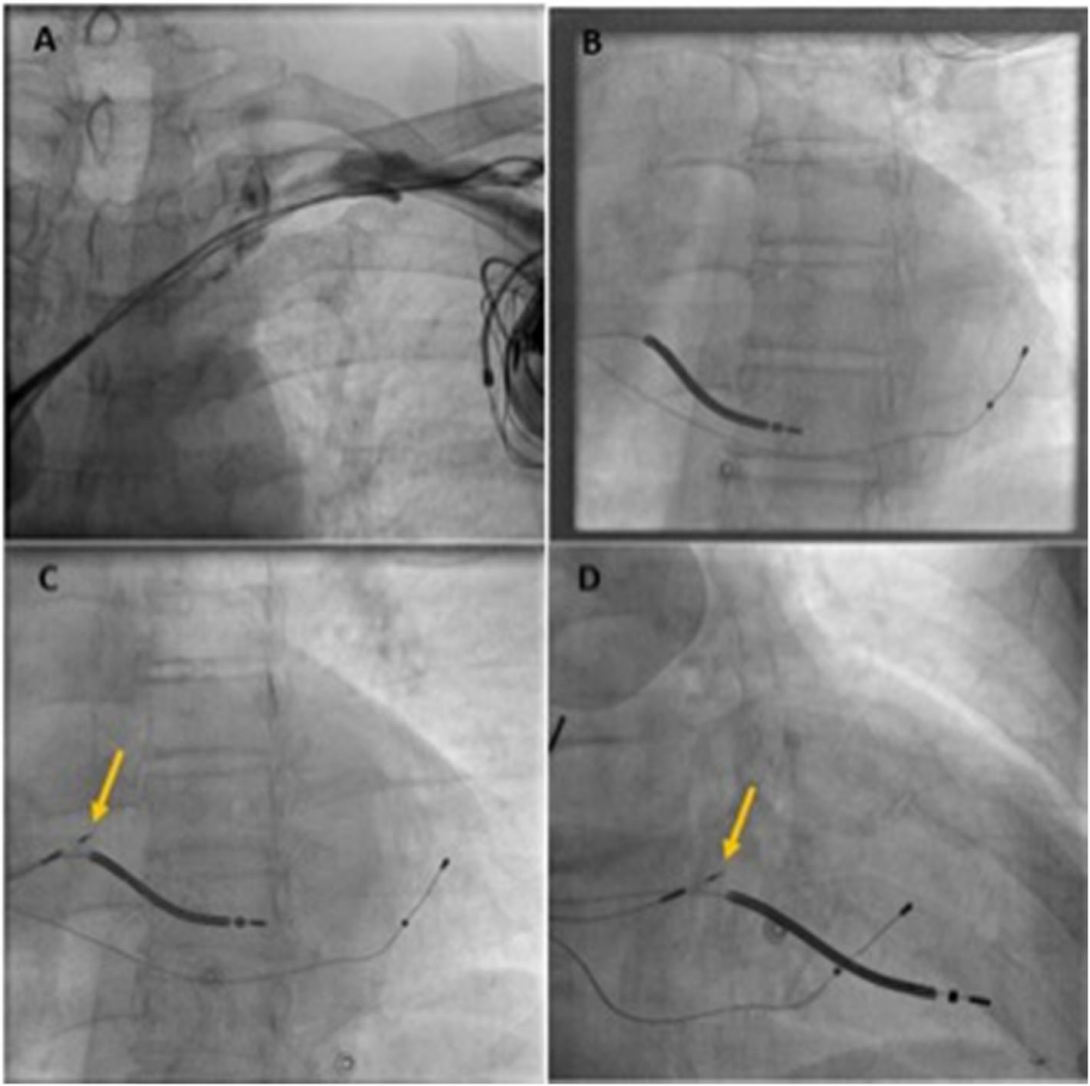
**a** Significant narrowing at left brachiocephalic and superior vena cava junction seen. **b** Placement of RV defibrillator lead and LV lead from the previous CRT implant. **c**, **d** Placement of new HBP lead in LAO & RAO view. The lead was given a 5–6 clockwise turn to fix at HB

**Fig. 4:**
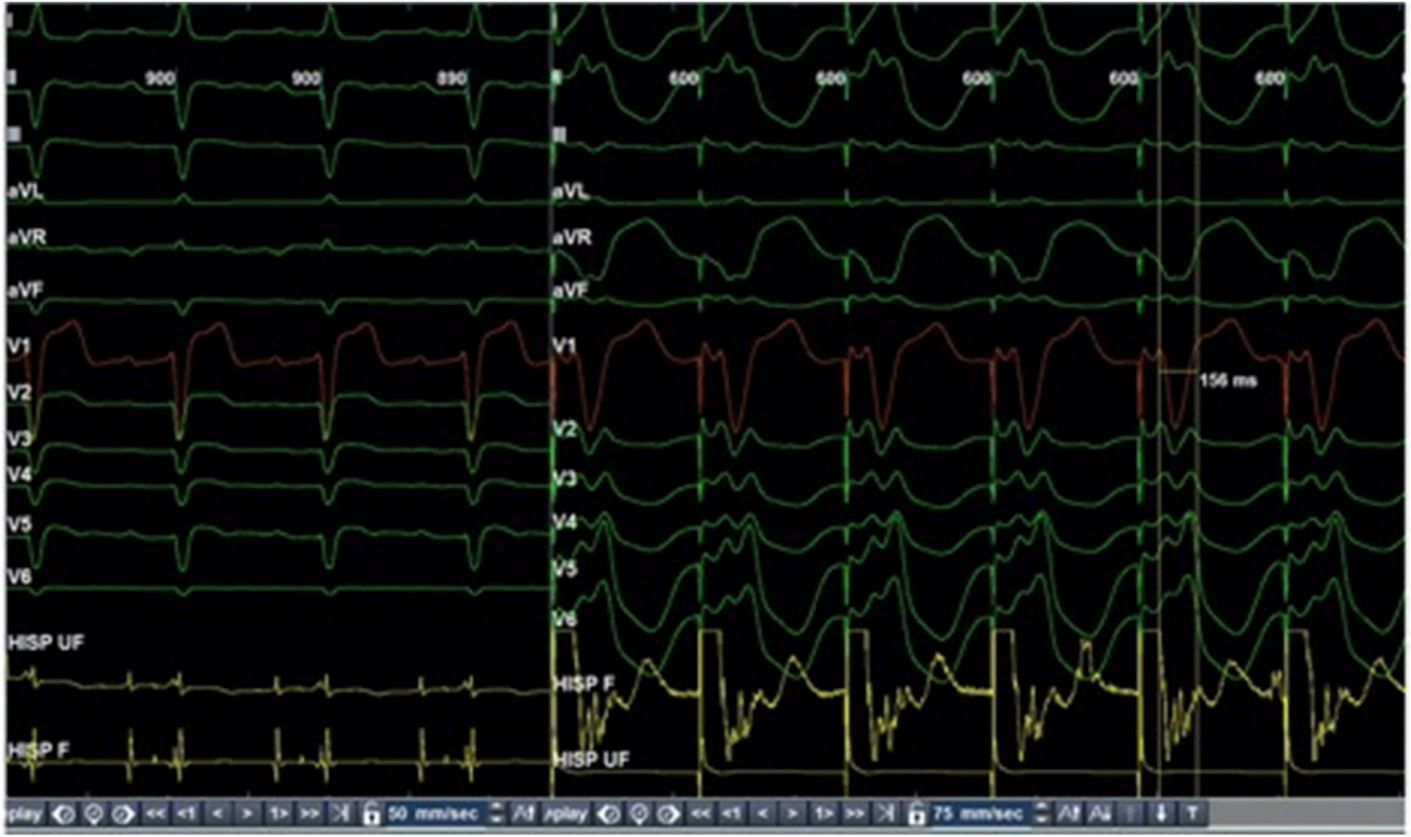
Distal His position with large ‘A’, wide paced QRS 154 ms and high threshold

A left axillary/subclavian venogram was taken from peripheral access vein to confirm patency. A significant narrowing at left brachiocephalic and superior vena cava (SVC) junction was seen (Fig. 3 a). A fluoroscopy-guided left subclavian access was obtained using Seldinger technique. A slippery Terumo wire was passed through the venous narrowing and access secured. The previous device was removed and the old leads parameters checked and secured. A fixed-curve sheath (C315 His, Medtronic Inc., Minneapolis, MN) was advanced over long Teflon wire into the right ventricle (RV). The Select Secure lumen less 4.1-F sized, 69-cm length 3830 SelectSecureTM active pacing lead (Medtronic Inc., Minneapolis, MN) was taken into the sheath. The lead was connected to Workmate Claris EP system for intracardiac electrogram (EGM). C315 catheter was pulled back into right atrium (RA) and turned anticlockwise to align it along the upper tricuspid annulus/RA junction. Local EGM showed His bundle potential in unipolar configuration. Pacing was done 5V @ 1 ms, resulting in nonselective HBP pacing. The threshold was 2.5/1 ms. Distal His position was tried but the threshold remained high (Fig. 4). Therefore LBBP was considered alternative and was attempted. The C315 sheath was advanced over the Teflon wire into the apex of right ventricle (RV) in right anterior oblique (RAO) projection along an imaginary line between the His bundle (HB) and RV apex using a road map of initial position of HB. The C315 sheath was positioned along the interventricular septum, 1–1.5 cm below the HB position but the lead could not be screwed into the left bundle as there was reverse transfer of the torque. Keeping in view the possibility of basal septal scar, posterior fascicle pacing was attempted by targeting the mid and posterior septum. Up to four sites including a distal part of septum were tried but lead did not advance beyond the initial one or two turns. In view of the possibility of tissue in helix of lead, it was cleaned of tissue bites after every attempt. Challenge was predominantly reaching mid-myocardial. Some maneuvers were done to let lead jump across the mid-myocardial scar and fall into LBB area like giving rapid turns with some force on the sheath but were not successful. After the failure to achieve LBBP, we returned to mapping the HB region, in search of a better pacing threshold. With some effort, we could find a spot with good local HB potential below the tricuspid valve with a pacing threshold of 1.7 @ 1 ms. R wave obtained at this position was 9 mV. The lead was given a 5–6 clockwise turn to fix at HB (Fig. 3c, d).

The HB lead was attached in RV pacing port (DF1 RV lead), coronary sinus (CS) lead in LV port, and RA lead in atrial port in the new pulse generator. Previous RV pace-sense lead terminal was buried deep in the pocket. The pre-pectoral device pocket was closed in three layers; DFT (defibrillation threshold test) was not done as per protocol.

**Fig. 5:**
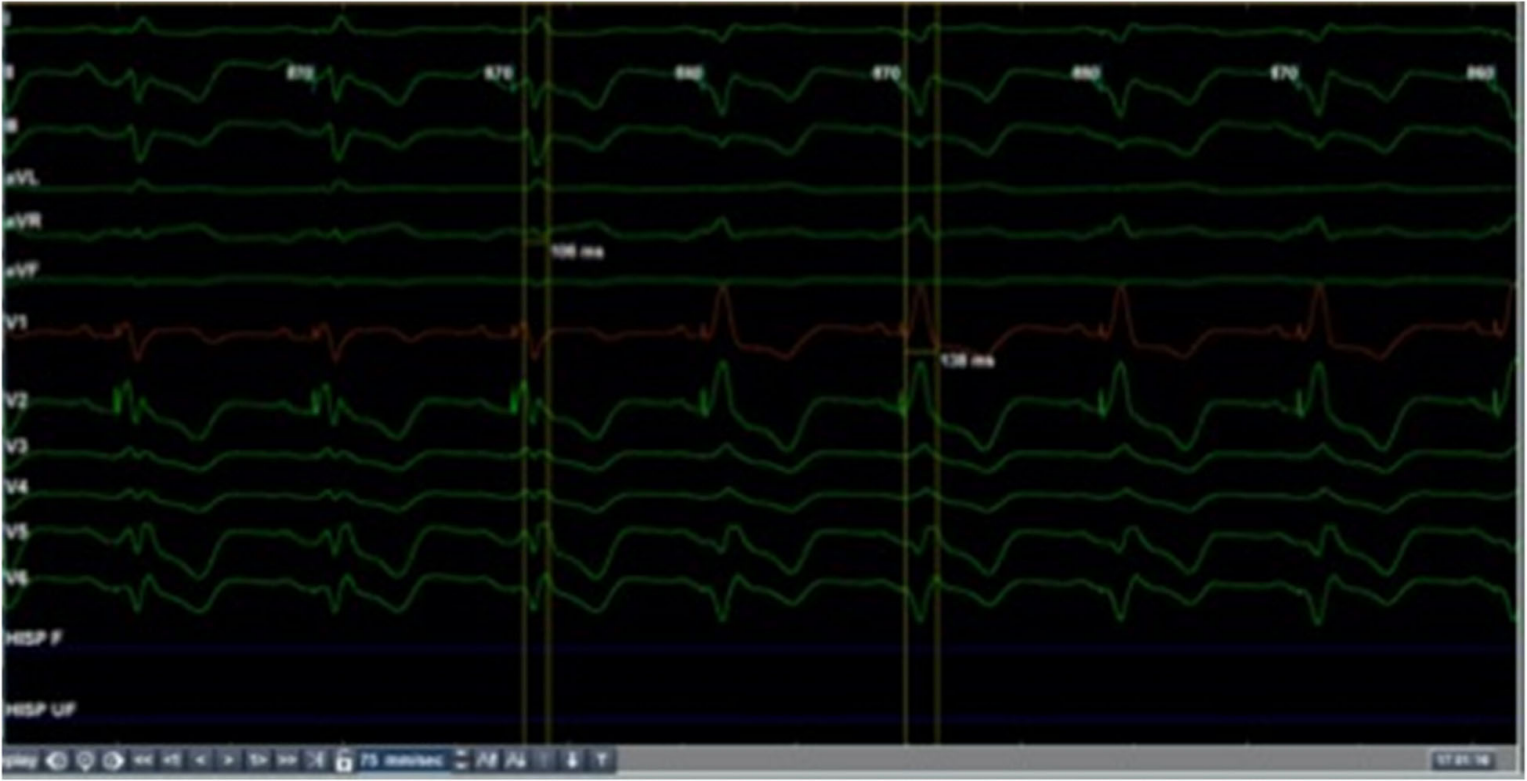
QRS resulting with HBP + LV pacing (106 ms) and only LV pacing (138 ms)

The strategy of fused complex was employed whereby LV pacing timing was delayed, relative to HBP output (Fig. 5). A narrow (106 ms) complex QRS was achieved with an AV delay of 80 ms and HBP-LV delay of 40 ms (Fig. 2b). QRS resulting from HBP + LV pacing (106 ms) and only LV pacing (138 ms) is shown in Fig. 5. At 6 months follow-up, patient showed improvement to functional NYHA class I-II and 2D echo showed LVEF 30% with PASP of 25 mm of Hg.

## Discussion

**Fig. 6:**
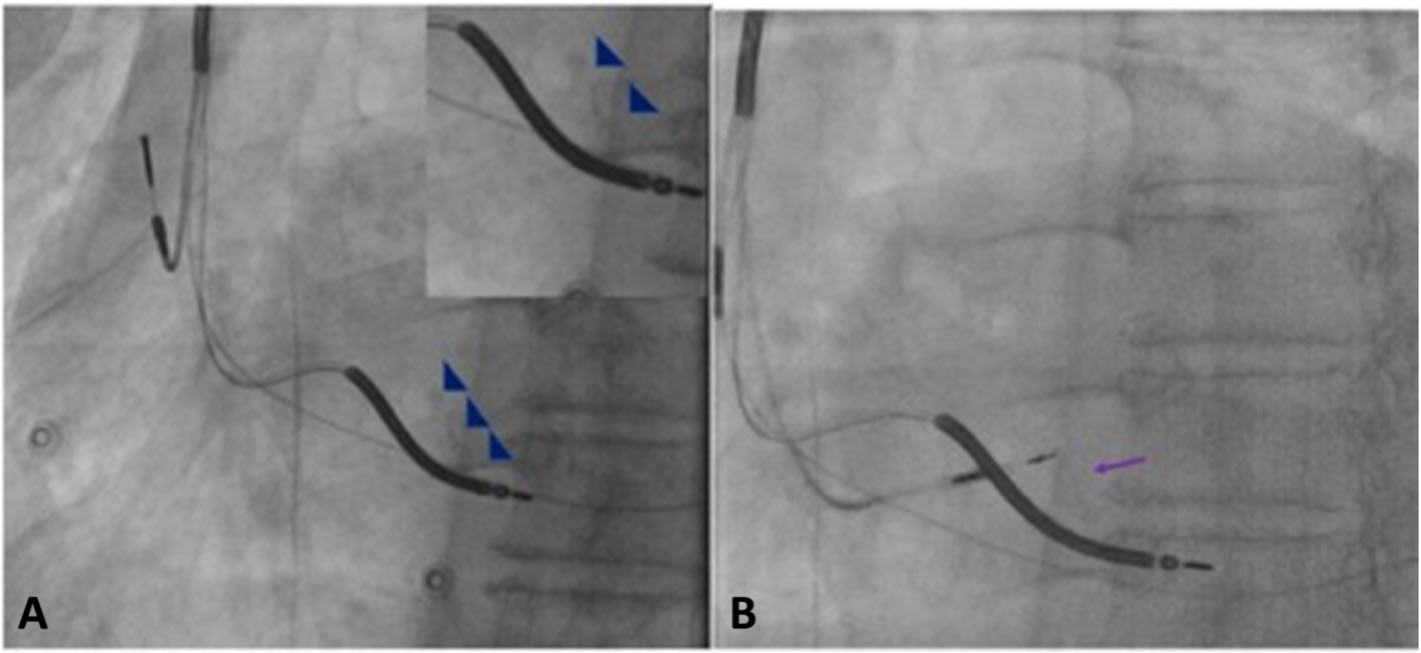
**a** Extensive calcification of septum (highlighted by blue arrows), due to which the clockwise torque on the lead was not being transmitted forward for penetration (**b**), instead was coming back causing the lead to make multiple turns and get entangled

About 30–50% of patients with HF who meet the criteria do not benefit by CRT [1]. In addition, the evidence for CRT in patients with non-specific IVCD with wide QRS and RBBB is sparse with conflicting results [2, 3]. However, a recent demonstration of significant narrowing of QRS duration and improvement in LVEF in patients with RBBB by HBP has paved the way for such technique in these groups of patients as well [4]. With this premise, we attempted physiological pacing using HOT-CRT strategy. In this case interestingly, we encountered a very rare but not yet reported limitation of the current hardware for LBBP. We failed to penetrate the mid-myocardial septum to reach the LV side of the septum to selectively pace the LBB. The reported success rate of LBBP in various reports is between 80.5 and 93% [5]. The reasons for failure are inadequate sheath support, improper sheath-septum orientation, failure to penetrate the lead deep into the septum, tissue lodged in the helix, septal scar, or entanglement of septal tricuspid leaflet. It is important to orient the sheath perpendicular to the septum and maintain the counter clockwise torque on the sheath during lead placement (hub must point towards 3’O clock position) [6]. This challenge can happen with interstitial fibrosis or scar or thickness or unusual orientation in dilated heart or calcification of septum. In our case, it was extensive calcification, due to which the clockwise torque on the lead instead of being transmitted forward for penetration was coming back causing the lead to make multiple turns and get entangled (Fig. 6a, b). This experience highlights the importance of assessing the interventricular septum (IVS) anatomy by echocardiography, fluoroscopy, or may be cardiac MRI before proceeding with the case.

## Conclusion

Through this case, we demonstrated that HOT-CRT is effective in correcting IVCD. It is an option particularly in patients who are CRT non-responders. Success of LBBP is dependent on IVS anatomy. Local dense scar or calcification at the septum may result in failure of LBBP. The present 3830 SelectSecureTM active pacing lead (Medtronic Inc., Minneapolis, MN) used for LBBP has limited penetration capability. Focused hardware needs to be developed to overcome such impediment.

## Data Availability

Yes, with the corresponding author in spreadsheet format

## Abbreviations

CRT: Cardiac resynchronization therapy
HF: Heart failure
LVEF: Left ventricular ejection fraction
HBP: His bundle pacing
LBBP: Left bundle branch pacing
BiVP: Biventricular pacing
HOT-CRTD: His-optimized CRTD
IVCD: Intraventricular conduction delay
PASP: Pulmonary artery systolic pressure
SVC: Superior vena cava
RV: Right ventricle
RA: Right atrium
EGM: Electrogram
HBP: His bundle pacing
LBBP: Left bundle branch pacing
LV: Left ventricle
RAO: Right anterior oblique
HB: His bundle
CS: Coronary sinus
HOT-CRT: His-optimized CRT
IVS: Interventricular septum
